# Trials of large group teaching in Malaysian private universities: a cross sectional study of teaching medicine and other disciplines

**DOI:** 10.1186/1756-0500-4-337

**Published:** 2011-09-09

**Authors:** Susan Thomas, Shamini Subramaniam, Mathew Abraham, LaySan Too, LooSee Beh

**Affiliations:** 1 PhD Student from University Malaya and Lead Coordinator School of Medicine Education Unit, Jeffrey Cheah School of Medicine and Health Sciences, Monash University, Sunway Campus, Jalan Lagoon Selatan, 46150 Bandar Sunway, Selangor, Malaysia; 2 Engineering Faculty, UCSI University, Jalan Menara Gading, USCI Heights, 56000 Kuala Lumpur, Malaysia; 3 Nottingham University Business School, University of Nottingham, Malaysia Campus, Jalan Broga, 43500 Semenyih, Selangor Darul Ehsan, Malaysia; 4 Volunteer Project Investigator, School of Medicine Education Unit, Jeffrey Cheah School of Medicine and Health Sciences, Monash University, Sunway Campus, Jalan Lagoon Selatan, 46150 Bandar Sunway, Selangor, Malaysia; 5 Faculty of Economics & Administration, University of Malaya, 50603 Kuala Lumpur, Malaysia

## Abstract

**Background:**

This is a pilot cross sectional study using both quantitative and qualitative approach towards tutors teaching large classes in private universities in the Klang Valley (comprising Kuala Lumpur, its suburbs, adjoining towns in the State of Selangor) and the State of Negeri Sembilan, Malaysia. The general aim of this study is to determine the difficulties faced by tutors when teaching large group of students and to outline appropriate recommendations in overcoming them.

**Findings:**

Thirty-two academics from six private universities from different faculties such as Medical Sciences, Business, Information Technology, and Engineering disciplines participated in this study. SPSS software was used to analyse the data. The results in general indicate that the conventional instructor-student approach has its shortcoming and requires changes. Interestingly, tutors from Medicine and IT less often faced difficulties and had positive experience in teaching large group of students.

**Conclusion:**

However several suggestions were proposed to overcome these difficulties ranging from breaking into smaller classes, adopting innovative teaching, use of interactive learning methods incorporating interactive assessment and creative technology which enhanced students learning. Furthermore the study provides insights on the trials of large group teaching which are clearly identified to help tutors realise its impact on teaching. The suggestions to overcome these difficulties and to maximize student learning can serve as a guideline for tutors who face these challenges.

## Background

The oldest conventional method of transferring information to a sizeable audience is very much instructor centred and makes learning passive therefore the need to reconsider the way courses are taught primarily due to the rapid increase in class sizes [1,2]. Classroom interaction can become depersonalised when the tutor has difficulties in establishing a more individualized tutor-student relationship [3]. It is not uncommon to have 400 students in a class now, compared to ten years ago when a class had 20 people and discussions are possible [4]. However for this study we will assume a large class as more than 80 students.

The conventional method of teaching large groups has been criticized as being negative as it encourages rote learning and there is a lack of independent learning by students. Teaching large groups is in danger of becoming a simplistic drive for pacification of the audience and simplification of the materials [4]. Whether tutors adopt the concepts "control" which means the learning remains with the tutor and "independence" which implies students' have more say in the learning, the teaching and learning challenges faced could ultimately lead to a disconnect in the student-teacher relationship [5]. It is impossible to give personal attention to students in these large groups as the reserved ones may tend to keep quiet, further limiting effective class interaction not only among students but also with the tutor who has the added burden of teaching in inadequate classroom facilities and environment [6-8].

Students express dissatisfaction and discontentment with the learning experience, when there is limited student-instructor interaction and the frustration is not only evident among students but also tutors when they are unable to relate to students as individuals [9,10]. However some believe teaching large classes or small classes is not an issue as the teachers' method and style would remain the same, rather the emphasis should be in changing teaching approaches [11,12]. Although this was peculiar in a primary school, it may not reflect the trials a tutor faces when teaching large groups in a university setting. It is also argued that large classes are crucial for "teaching independent learning and communications skills because they set the tone for a student's whole academic career" [13].

In enhancing student learning, interactive lecturing promotes active involvement, increased attention and motivation, and increases the satisfaction of both teachers and students in medical education [14,15]. Furthermore effective learning should include: inquiry-based and problem-based learning and implementing smaller group activities [16-18]. An action learning approach not only would significantly impact the pedagogy, teaching strategies and course design but also lead to effective action where learners connect, and take responsibility in solving identified problems [19]. The problem based learning curriculum in medicine has gained strong interest and serves as a way to make knowledge more relevant and retrievable, and to foster the development of specific reasoning [20]. The anguish of stimulating desire and constantly motivating students to learn using creative teaching is a big challenge for tutors and there is a deep need to reflect on this.

Therefore the aims of this study are to:

a. determine the teaching difficulties and anguish faced by tutors from school of Medicine and other disciplines.

b. identify the teaching resources used by tutors who teach large group of students.

c. determine how students' learning experience can be enhanced

d. review the effect of teaching difficulties and anguish faced by tutors from medicine and other disciplines

e. provide recommendations to overcome teaching difficulties.

## Methods

### Participants

This study involved the participation of academics from various foreign and private universities. Heads of Departments and Research Directors of each faculty of the universities were approached individually to explain about the purpose of this research, to find out about programmes that are taught in large numbers and also to seek approval. Numerous correspondences via e-mails, short messaging systems and telephone calls were made to the various institutions to follow-up with the research projects. Six private Universities from the central urban area of Klang Valley in Malaysia consented to participate in this study and to disseminate information and anonymous questionnaires to their academic staff. Participation was on a voluntary basis and the questionnaires returned were considered as implied consent.

Thirty-two participants volunteered to take part in this study. In terms of gender distribution there were seventeen male and fifteen female tutors. Out of this, fourteen tutors were from School of Engineering, eight from School of Business, six from School of Medicine and Sciences and four from School of Information Technology.

### Materials

A pre-test was conducted on a small group of academicians who were involved in large group teaching before the actual distribution of the questionnaire. The purpose was to test the reliability and improve the quality of the questions designed in the questionnaire. The final version of the questionnaire was designed using a series of twelve semi-structured questions that are divided into Part A and Part B (see Appendix 1). The questions in Part A were used to gather information regarding the teaching background of the tutors, number of years of teaching experience, the pedagogy used, the size of class and contact hours. Questions in Part B were used to gather information regarding the major trials faced by the tutors in teaching large groups and how it affects them using 5-points Likert scale (1-Never, 2-Rarely, 3-Sometimes, 4-Most of the time, 5-Always). In part B, the approaches tutors use in overcoming difficulties and how the students' learning experience can be enhanced were gathered utilising open-ended questions. An overall rating is also included at the end of the questionnaire for the tutors to analyse their experience with 5 choices given (strongly disagree, disagree, neutral, agree & strongly agree). Reliability analyses were also undertaken, showing a high Cronbach's alpha of 0.84 for 17 items of problems faced in large group teaching. Moderate Cronbach's alpha of 0.69 for 5 negative items (e.g. added stress or anxiety, feel mentally exhausted, physically tired, compromise on personal time with family/close friends, poor student evaluation) and 0.75 for 5 positive items (e.g. gives me a sense of achievement, feel good and cheerful after the class. Other statement such as like the challenge, allows me to adapt to my students' learning needs, makes me more aware of my teaching styles) were demonstrated on the scale examining effects from large group teaching.

### Design, Procedure and Limitations

This study obtained ethics approval from USCI University research committee. The primary method of distribution of questionnaire was to distribute personally to the tutors.

Descriptive analyses were used for the majority of the data analysis because of the small sample size. As compared to the number of tutors from non-medicine disciplines (i.e. Business, Information Technology, and Engineering) (n = 26), only a fraction of tutors from medicine discipline (n = 6) participated and included in the study. This big discrepancy with the number of tutors between medicine and non-medicine groups would make statistical comparisons a difficult task to achieve. Therefore, only a brief overview and discussions on the quantitative results were presented. An arbitrary cut-off criterion of mean equal to three (on a five-point scale) and above was used to determine the most significant problems or effects faced by the majority of participants while less significant problems or effects were identified using an arbitrary cut-off criterion of mean less than three. Further analysis was made based on the qualitative results received on 'how students' learning experiences can be enhanced' and the 'recommendations to overcome difficulties'.

The main limitation in this study is finding suitable tutors who teach large groups of students and getting them involved in this study, given the busy schedule they have. Furthermore there were also difficulties in seeking permission from various Universities and Colleges in Klang Valley to participate in this research. These institutions were unfamiliar with the benefits of collaborative research especially in the educational sector in Malaysia. Many of them have no clear policies in data collection and research ethics. Some of the universities have had a change in management and were sceptical in participating. They were also not certain as to who should approve the research exercise. Is it the CEO, Research Director or Head of Department?

As a result there were numerous red tapes that had to be faced which required a large amount of time in networking and convincing them to be involved in this pilot study. Furthermore, not all the faculties offered courses that had large group of students. Thus a further screening is required before questionnaires were distributed to the right group of tutors.

## Results

The teaching demographic characteristics of participants are outlined in Table 1. In terms of the teaching experience, the majority of the tutors (n = 22) have less than 4 years of teaching experience. Only four tutors have teaching experience that exceeded 10 years.

**Table 1:** Teaching demographic characteristics of participants

Demographic	Participants number (n = 32)	Participants%
*Faculty/Department*		

Medicine	6	18.8
Engineering	14	43.8
Business	8	25.0
Information technology	4	12.5

*Gender*		

Male	17	53.1
Female	15	46.9

*Years of Teaching*		

< = 1	11	34.4
2-4	11	34.4
5-7	4	12.5
8-10	2	6.3
> 10	4	12.5

*Largest Group Size*		

80 - 100	13	40.6
101 - 150	8	25.0
151 - 200	4	12.5
201 - 250	3	9.4
251 - 300	3	9.4
> 300	1	3.1

*Average Contact hours per Class*		

1.0	2	6.5
1.5	12	38.7
2.0	10	32.3
2.5	0	0.0
3.0	4	12.9
4.0	3	9.7

In terms of class size twenty-five tutors had the largest class size consisting of 80 to 200 students, six tutors had a class size of 200 to 300 students while only one tutor had a class size of more than 300 students. Twenty-two tutors had an average of 1.5 to 2 hours of contact times per class, followed by four of them with 3 hours contact time and three of them with 4 hours of contact time. Only two tutors had an average of 1 hour of contact time per class.

**Figure 1:**
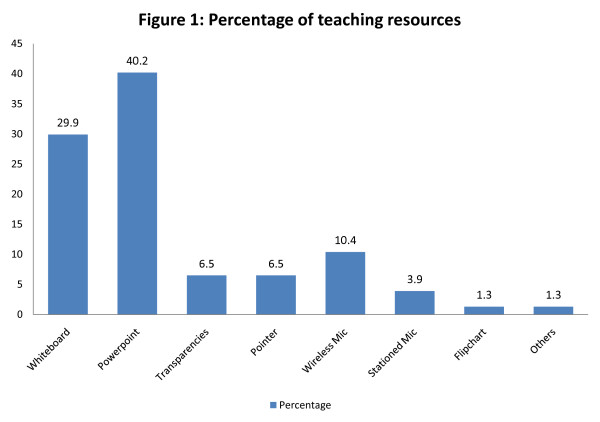
**Percentage of teaching resources**.

Results show that resources such as power-point slides were most frequently used in large group teaching (40.2%), followed by whiteboard (29.9%). However, the teaching resources which were less often used were wireless microphone (10.4%), pointers (6.5%), transparencies (6.5%), and stand/stationed microphone (3.9%). Flipchart and media player were rarely utilised for large group teaching in which they only accounted for 1.3% respectively. Figure 1 illustrates the percentage in terms of the usage of teaching resources.

### Problems of large group teaching: Across disciplines

The descriptive analysis showed that the most frequent problems faced by tutors in large group teaching were ensuring students are paying attention (M = 3.59, SD = 0.84) and difficulty in assessing tests, quizzes and assignments (M = 3.52, SD = 1.10). The tutors indicated that they were often confronted with problems in getting students to interact/participate (M = 3.34, SD = 0.79) and in identifying weak students (M = 3.31, SD = 0.90) (refer to Table 2).

**Table 2:** Problems faced in large group teaching

**No**.	Problems	Mean	Std. Dev
E.	Ensuring students are paying attention	3.59	0.84
Q.	Difficulties in assessment of tests, quizzes and assignments	3.52	1.09
H.	Getting students to interact/participate	3.34	0.79
B.	Identifying weak students	3.31	0.90
D.	Building relationship with students	3.19	0.97
A.	Maintaining discipline in the classroom	3.16	0.72
N.	Inability to be sensitive to students' learning needs	3.13	0.83
L.	Gauging students' understanding of the lesson	3.09	0.86
G.	Creating enthusiasm in class	3.03	0.82
M.	Managing students from different backgrounds/nationalities	3.03	1.00
O.	Unable to get more creative in your teaching methods	3.00	0.88
P.	Having too many subjects to teach per semester	2.97	0.97
C.	Bright students are sufficiently challenged	2.94	0.84
I.	Achieving learning objectives within the given time frame	2.84	0.95
F.	Ensuring finishing class/syllabus on time	2.69	1.26
J.	Lack of appropriate technology/facilities	2.69	1.09
K.	Audibility of your voice	2.41	1.13

As revealed in the descriptive analysis, audibility of voice of the tutors (M = 2.41, SD = 1.13) was the least likely to happen in large group teaching, followed by ensuring finishing the class/syllabus on time (M = 2.69, SD = 1.26) and lack of appropriate technology/facilities (M = 2.69, SD = 1.09). It is interesting to note that one tutor indicated that managing exams for large groups were a problem; a comment not fed back by other tutors.

### Effects of large group teaching across disciplines

The data derived from the open-ended questions indicated that tutors from IT and Medicine had positive experience from teaching student in large group setting. The feedback given by them showed that they have a sense of achievement because they felt challenged in handling a large class. Furthermore, teaching a large class also created a sense of awareness of their teaching styles in which they were able to adapt to the different learning styles of their students.

However, the results from the open-ended questions have also derived some of the unfavourable effects faced by tutors in teaching large group. The findings show the most unfavourable effect confronted by tutors from all the other disciplines were feeling mentally exhausted (M = 3.22, SD = 1.01), followed by physically tired (M = 3.19, SD = 1.12). Whereas, the impact of compromising on personal time with family/close friends (M = 2.56, SD = 1.08) was the least reported by the tutors (refer to Table 3).

**Table 3:** Effects of large group teaching

**No**.	Effects	Mean	Std Dev
	*Negative effects*		
B.	Feel mentally exhausted	3.22	1.01
C.	Physically tired	3.19	1.12
A.	Added stress or anxiety	2.97	1. 00
E.	Poor student evaluation	2.81	0.79
D.	Compromise on personal time with family/close friends	2.56	1.08
	*Positive effects*		
F.	Gives me a sense of achievement	3.25	0.95
G.	Feel good and cheerful after the class	3.03	0.93
H.	Like the challenge	3.53	0.95
I.	Allows me to adapt to my students' learning needs	3.00	0.67
J.	Makes me more aware of my teaching styles	3.63	0.91

### Recommendations to overcome teaching difficulties

The recommendations (Table 4) to overcome difficulties faced by tutors in large group teaching were coded into 14 categories. The participants were able to provide more than one recommendation for this section. The analyses showed that smaller teaching groups (35.7%) was the most favoured class size. There were also strong support in using innovative teaching methods (16.2%). Other important proposals given by tutors was on time management whereby 14.4% suggested breaks between classes, giving more time for preparation and keeping lecture content precise which would help overcome the difficulties faced in large group teaching. There were also strong emphases (3.6%) on the need for strong support from management when tutors faced these difficulties. Other recommendations (each 1.8%) include increasing manpower, extending the semester, explaining subject's objective, explaining the importance of the subject, giving support by providing better facilities and providing hands-on exercise. It is ironic that the tutors themselves realize the importance of incorporating innovative ways to enhance teaching and make them a meaningful learning experience for students. However the issue in question is; how do they make this happen in class and why don't they do it?

**Table 4:** Recommendations to overcome difficulties faced in large group teaching

Recommendations	N	%
Smaller group size	20	35.7
Innovative teaching	9	16.2
Time management	8	14.4
Interpersonal relations with students	4	7.2
Teaching through experience	3	5.4
Spread out teaching load	3	5.4
Support from management	2	3.6
All other suggestions (< 2%)	6	10.8

### Recommendations for enhancing students' learning experience

Qualitative data on ideas (Table 5) to enhance students' learning experience was categorised into twenty-three statements. Participants were able to provide more than one recommendation. The analyses showed that interactive learning method (18.0%), creative assessment (14.0%), use of technology (10.0%), smaller group size (10.0%), and hands-on experience (8.0%) were most often suggested. Some of the tutors indicated that giving consultation time for students (4.0%) and self-directed learning (4.0%) could enhance students' learning experience. Other suggestions proposed which constitutes 2.0% each consisted of preparation, evaluation of teaching delivery, break between classes, allocation of time for questions and answers, revision of topics, forming study group, giving handouts, increasing teaching hours, making tutorials compulsory, designing syllabus effectively, conducting industries trips, exploring more than one textbook for a particular topic, providing better facilities, inviting guest speakers, including more training and problem based learning respectively.

**Table 5:** Recommendations to enhance student's learning experience

Ideas	N	%
Interactive learning method	9	18.0
Creative assessment	7	14.0
Use of technology	5	10.0
Smaller group size	5	10.0
Hands-on experience	4	8.0
Consultation time for students	2	4.0
Self-directed learning	2	4.0
All other suggestions (2%)	16	32.0

### Tutors' overall experience in teaching large group

The final question in the questionnaire included an overall statement about the tutor's experience in teaching large group in their university and if change is required, were coded in 5-points scale (1-strongly disagree, 2-disagree, 3-neutral, 4-agree, 5-strongly agree). The overall analysis demonstrates that thirteen participants (41.9%) disagreed that large group teaching conducted in their University worked well with them and felt that change is required. On the other hand, ten participants (32.2%) agreed their experience in teaching large group worked well with them and did not require any changes. However eight participants (25.8%) did not have any opinion on this as they were neutral in their position.

### Tutors' overall experience of large group teaching: Across disciplines

In terms of overall experience of large group teaching across disciplines, tutors from school of IT (M = 3.67, SD = 0.58) and Medicine (M = 3.33, SD = 0.82) agreed that the overall experience in teaching large groups in the university fits them well and it does not require any specific changes. However, tutors from the Business school (M = 3.00, SD = 0.93) were neutral and did not have any opinion on this whereas tutors from Engineering (M = 2.29, SD = 1.20) disagreed with this statement.

## Conclusion

It is evident that majority of the tutors agreed that teaching large groups using the conventional instructor-student approach does not work well with them and it requires changes. However, tutors from schools of IT and Medicine respectively hold opposite opinions. The findings from the open-ended questions gathered from the questionnaire shows that the tutors from Medicine and IT often face less difficulties in teaching large group of students. In fact, most of them have indicated that they experience a positive effect as a result of teaching their large class because it provided them a challenge and gives them a sense of achievement. Whereas the tutors from the other disciplines felt mentally and physically tired after teaching a large group. This means the need to adopt different teaching methods across different disciplines. The common and greatest problem for tutors in teaching large groups is to ensure students remain attentive in class, the difficulties in implementing appropriate assessments and to get students to participate or just talk. Some of the changes recommended to overcome these difficulties are breaking into smaller classes and using innovative teaching. Further enhancement towards learning would be the use of interactive learning methods such as introducing interactive assessment and creative technology. However, most importantly tutors view large group teaching as a challenge rather than a weakness. Although it may create some degree of fear and expose flaws in the actual teaching style but when conducted effectively it may actually create a sense of self-esteem and self-actualisation.

The evidence from previous studies strongly suggests large group teaching affects tutors emotionally and lead to problems. Therefore a way to reduce these emotional effects were the recommendation to use interactive teaching and learning methods which would enhance student learning especially when tutors teach large groups of students.

## Recommendations

This pilot cross sectional study not only provides insights on the trials faced by tutors when teaching large groups but also provides a greater sense of awareness in the teaching approach adopted across different disciplines. The comparison also highlights the different set of challenges across disciplines and the different teaching approaches used for large group teaching. The recommendations can serve as best practice encompassing issues on teaching approaches and in providing a conducive teaching environment to enhance student learning experience.

### Optimum class size

Universities and to a lesser extent relevant monitoring agencies should relook at the optimum class size and if necessary implement guidelines on this. A limit to the number of students per class would facilitate the process of interactive learning as supported by both the present and previous studies. Having large classes makes sense in the financial sense but its implications to tutors and the students learning experience must be given serious thought. Tutorials should therefore consist of limited number of students which facilitates student engagement and interactive learning.

### Sharing of teaching experiences

The mutual sharing of positive teaching experiences is an important element as it promotes positive and enjoyable teaching spirit among tutors. Most of the time teaching large group gives tutors a sense of achievement, a sense of challenge and an innate awareness of one's own teaching styles. Does this mean the traditional lecturing will die a slow death and tutors become radical advocates of creative and transformational teaching? This will not happen as many will continue to use it as the most convenient way to impart knowledge. Tutors with many years of teaching experience should share insights on good teaching practices with beginner tutors on ways to improving teaching methodology and provide an enriching learning experience to students.

### Teaching aids

This study reiterates the fact that tutors generally lack creativity and innovation in their pedagogy. Most cite the commonly used resource is the power-point (42%) but it still remains as a very small percent. However it is emphasized that "no one delivery style is optimal for all content and context" and "the blind use of a particular presentation" is not encouraged therefore clearly the "intelligent use" of power point is important to ensure effective delivery and retention of lecture content by students [21]. A good practice is to provide lecture hand-outs or up-load them on-line, one day before the lecture to inculcate the importance of preparation for an interactive learning process. To elicit a wider span of attention it would be stimulating to involve students through questioning and praising them for their effort [22]. Tutors today can integrate a variety of innovative teaching resources ranging from podcasting, student response system, interactive based media when delivering lectures which ultimately can be an extremely enriching and satisfying learning experience for students.

### Teaching activities

It is important to overcome the emotional disconnect between tutor and student so that large group teaching is not burdensome but a catalyst for developing deeper insights into creative and innovative teaching that is emotionally satisfying for both. The learning outcomes of the lectures in large classes can then be further reinforced in tutorials and practical sessions. Tutors can overcome any disconnect between the tutor and student by adopting situational, action or problem based learning in their teaching pedagogy. The ability to transfer existing knowledge gained from lectures and applying them to new contexts and situations is part of the process of situational learning [22]. Similarly in business modules, the use of case studies teaches students to think critically, evaluate business situations and make informed decisions from this evaluation. In a very large class tutors should incorporate small group activity during lectures to motivate and inspire students to learn and reiterate that the "listen and learn" approach is no longer desired [23]. Ultimately tutors need to encourage and develop students to be self directed learners who take initiative and responsibility in their own learning.

### Future study

Problems and effects faced by tutors in teaching large groups as well as ways to overcome the difficulties, and enhance student learning were identified using qualitative and quantitative methods. However, the limitations in this study are the small sample size in which the participants were recruited from especially the sample size of tutors from medicine. Besides limitations in the sampling size, there were also limitations in the sampling frame. Even though invitations were given out to all the private universities in Klang Valley, there were only about six private universities in Malaysia that agreed to take part in this study. This is because some of them had guidelines and restrictions that were difficult to follow and not all of the programs offered in these Universities conducted large group teaching. The researchers had to go to great lengths in meeting the various department heads with follow-up letters, e-mails and telephone calls to explain the purpose of this study and methods proposed for data collection. As a result, the duration in collecting data took longer than expected, which is about eight months. Another limitation to this study is that this research is concentrated in the urban areas of Klang Valley in Malaysia. It would be interesting to note the problems faced by tutors in other parts of the country.

Even though this study has a small sample size, it can provide reliable findings depending on sampling procedures undertaken [24]. Therefore this study can be used as a guide for bigger projects with involvement of larger number of participants from other states and from public universities. For future study, the correlation between different class size and the effect on tutor teaching experiences and its relationship to students' learning experiences could also be examined. This study mainly aims to determine whether class sizes have impact on teaching and also to identify ways to enhance teaching and learning experiences. For future research it would also be interesting to investigate if tutors with formal training on teaching have any impact in the way students are taught.

## Competing interests

The authors declare that they have no competing interests.

## Authors' contributions

ST conceived the idea, developed the questionnaire, corresponded and collected data from other universities, contributed to the analysis and wrote the manuscript. SS developed and distributed the questionnaire, obtained ethics approval and proofread the manuscript. MA collected the data and wrote the manuscript. LST performed statistical analysis, interpreted the data and wrote the manuscript. LSB reviewed the analysis and edited the manuscript. All authors have read and approved the final manuscript.

## Appendix 1 (Questionnaire - condensed version)

### A. Background

1. Gender

2. How long have you been teaching large group of students?

3. Which faculty are you in?

A. Engineering B. IT C. Business D. Medical Sciences

4. What is the size of your largest class?

5. What are the average contact hours per class?

6. What teaching resources do you normally use for a large group?

### B. Trials and the effect of large group teaching faced by instructors

7. What are the most significant problems you face teaching a large group?

Please rate as: (Never- 1, Rarely - 2, Sometimes - 3, Most of the time - 4, Always - 5)

A. Maintaining discipline in the classroom ____

B. Identifying weak students ____

C. Bright students are sufficiently challenged ____

D. Building relationship with students ____

E. Ensuring students are paying attention ____

F. Ensuring finishing class/syllabus on time ____

G. Creating enthusiasm in class____

H. Getting students to interact/participate ____

I. Achieving learning objectives within the given time frame ____

J. Lack of appropriate technology/facilities _____

K. Audibility of your voice _____

L. Gauging students' understanding of the lesson ____

M. Managing students from different backgrounds/nationalities ____

N. Inability to be sensitive to students' learning needs ____

O. Unable to get more creative in your teaching methods _____

P. Having too many subjects to teach per semester ____

Q. Difficulties in assessment of tests, quizzes and assignments _____

R. Others (please specify)____

8. How does teaching a large group affect you? Please rate as:

(Never- 1, Rarely - 2, Sometimes - 3, Most of the time - 4, Always - 5)

A. Added stress or anxiety ____

B. Gives me a sense of achievement____

C. Feel good and cheerful after the class ___

D. Like the challenge _____

E. Feel mentally exhausted ____

F. Allows me to adapt to my students' learning needs _____

G. Makes me more aware of my teaching styles_____

H. Physically tired ____

I. Compromise on personal time with family/close friends ____

J. Poor student evaluation ____

K. Others (please specify) ______

9. What do you recommend to overcome the above difficulties?

10. How else do you think the students' learning experience can be enhanced?

11. What are the benefits (if any) of teaching in a large group?

12. Overall, my experience in teaching large groups in this university works well with me and does not require any changes. (Please circle ONE).

A. Strongly Disagree B. Disagree C. Neutral D. Agree E. Strongly Agree
